# Safe Communities in China as a Strategy for Injury Prevention and Safety Promotion Programmes in the Era of Rapid Economic Growth

**DOI:** 10.1007/s10900-012-9594-4

**Published:** 2012-08-10

**Authors:** Shu-Mei Wang, Koustuv Dalal

**Affiliations:** 1 School of Public Health, Key Laboratory of Public Health Safety, Ministry of Education, Fudan University, Shanghai, China; 2 School of Health and Medical Science, Department of Public Health Science, Örebro University, 701 82 Orebro, Sweden

**Keywords:** Injury prevention, Safety promotion, Safe Community, Economic development, China

## Abstract

Due to its rapid economic development, China is facing a huge health, social, and economic burden resulting from injuries. The study’s objective was to examine Safe Communities in China as a strategy for injury prevention and safety promotion programmes in the era of rapid economic growth. Literature searches in English and Chinese, which included grey literature, were performed on the Chinese Journal Full-text Search System and Medline, using the words “Safe Community”, “injury”, “economics”, and “prevention”. The results showed that the existing 35 recognized members of the International Safe Community Network have not placed due emphasis on suicide prevention, which is one of the leading problems in both rural and urban China. A few groups, such as children, the elderly, cyclists, and pedestrians, have received due emphasis, while other vulnerable groups, such as migrant workers, motorcyclists, students, players, and farmers have not received the necessary attention from the Safe Community perspective. As the evidence describes, Safe Communities in China can be a very effective strategy for injury prevention, but four aspects need to be strengthened in the future: (1) establish and strengthen the policy and regulations in terms of injury prevention at the national level; (2) create a system to involve professional organizations and personnel in projects; (3) consider the economic development status of different parts of China; and (4) intentional injury prevention should receive greater attention.

## Introduction

Looking back at the work of disease control during the last 50 years in China, it can be divided into three stages as follows [1–4].

The first stage was the early years of the establishment of the People’s Republic of China when infectious, parasitic, and endemic diseases were rampant and China was facing a serious threat because of a variety of severe acute infectious diseases. Disease control focused on acute infectious diseases.

The second stage was manifested as a decline in the morbidity and mortality of infectious diseases and an increase in the elderly population; the threat to people’s health due to chronic non-communicable diseases gradually increased, and the emphasis of disease control since the 1980s included both infectious diseases and chronic non-communicable diseases, such as tumours, heart disease, hypertension, stroke, diabetes, and so on.

The third stage was the era of economic development, in which the degree of increased mobility and people’s fast pace of life was accompanied by the threat of injuries. In the 1950s, mortality due to injuries and accidental poisoning ranked ninth in the causes of death, in the 1970s it moved up to seventh place, while in the 1990s it rose to fourth place. Injury prevention became one of the focuses of disease control. The Safe Community programme was designed to control injuries and promote safety and has been evidenced to be effective [5, 6].

A set of figures indicates that China has progressed from being a low-income country into the ranks of middle-income countries. In the last 60 years, the GDP growth has exceeded 77 times with per capita GDP of over 3,000 US$. Revenue grew about 1,000 times. Foreign exchange reserves increased over 10,000 times, ranking as the first in the world. The import and export trade volume ranked third, accounting for 7.9 % of the world trade [7]. With the pace of economic growth and development, injuries have also increased almost at the same pace [8].

A World Bank report estimated that the number of injury deaths in China would reach 1.4 million in 2010 [9]. Especially in recent years, during which economics in China has developed very quickly, some kinds of injuries have been rising as well, such as traffic injuries, falls, suicide, etc.

Diversity in the nature of injuries and their risk factors are major challenges in setting appropriate injury prevention policies. However, effective injury prevention and safety promotion strategies have been evidenced for the past few years, especially in high-income countries. The main challenge is to adapt this knowledge to country-specific settings, in an effective, understandable framework. A well-designed injury prevention policy is invaluable. The policy should raise awareness and create understanding of the common goal of injury prevention and safety promotion.

How to control and prevent injuries that have brought great harm to Chinese people has become one of the top priorities for disease control in China. Safe Communities have been developed to control injuries and promote safety [5]. The effectiveness of Safe Community programmes in decreasing injury rates has been proved in high-income countries on a number of occasions [10–13]. Safe Communities are also economically effective. Studies from Europe have shown that with the Safe Community intervention cost of 1.5 million USD, the societal cost savings for injuries were 3 million USD (approximately) in the intervention areas [14]. The overall cost–benefit ratio is 1:10 for Safe Communities in industrialized countries [6]. The programme is being implemented worldwide. China has strongly emphasized the Safe Community movement for injury prevention and safety promotion. So far China has 35 designated communities as members of the International Safe Community Network. However, analyses of Safe Communities are lacking in the literature in China. The objective of the study is to examine Safe Communities in China as a strategy for injury prevention and safety promotion programmes in the era of rapid economic growth. To gain a better understanding of the issue, the risk factors and the burden of the leading injuries in the context of China are also studied.

## Methods

The following databases were searched for potential scientific articles: the Chinese Journal Full-text Search System and Medline. Google Scholar was also used for relevant grey literature support. The search terms used were “Safe Community”, “injury”, “economics”, and “prevention”. Statistics and reports of the Government of China, the WHO, and other international bodies were also included in the study. All research designs were accepted for inclusion if they met the following criteria: published in Chinese or English, containing data exclusively on injuries, focusing on primary prevention and safety promotion, and studying the population in China. Articles not focusing on China were excluded.

## Results

### Economic Development and Injury

Economic development has brought great changes to people’s daily lives. For example, in the transport sector, twenty or thirty years ago it was unimaginable for Chinese people to have a private car to commute back and forth from work or to travel in their own car. However, the data show that from 1985 to 2005 the number of motor vehicles in China increased 19 times, and the rate of motor vehicle ownership per thousand people rose 15 times. Taking Shanghai as an example, in 2000 the motor vehicle ownership per thousand people was 78.9, while in 2002 the figure was 106.7 (more than 50 % over a two-year period) [15].

The estimated annual economic cost of injuries is equivalent to 12.5 billion USD, which is almost four times the total public health services budget of China [16]. According to data from the Ministry of Public Security, at the end of 2009 the number of motor vehicles in China reached 186 million and the number of drivers was almost 200 million. While a higher degree of motorization in transportation brought convenience to the people, it also showed an increase in traffic accidents year by year [17]. Road traffic mortalities have increased along with the economic development in China. Almost one-quarter of the total potential productive years of life lost (PPYLL) from all injury deaths are due to traffic injuries in China [16].

From 1951 to 2002 road traffic injuries have continued to increase in China; especially after the 1980 s the trend became more significant, mainly regarding the number of deaths due to motor vehicle traffic accidents. Since 2000 the number of traffic fatalities every year has been around 100,000, while the number of injuries has been around 500,000. There has been a hundredfold increase in road traffic mortality during the past 55 years [18].

Worldwide, road traffic accident deaths are projected to increase from 1.3 million in 2004 to 2.4 million in 2030, primarily due to the increased motor vehicle ownership and use associated with economic growth in low- and middle-income countries [19].

**Fig. 1:**
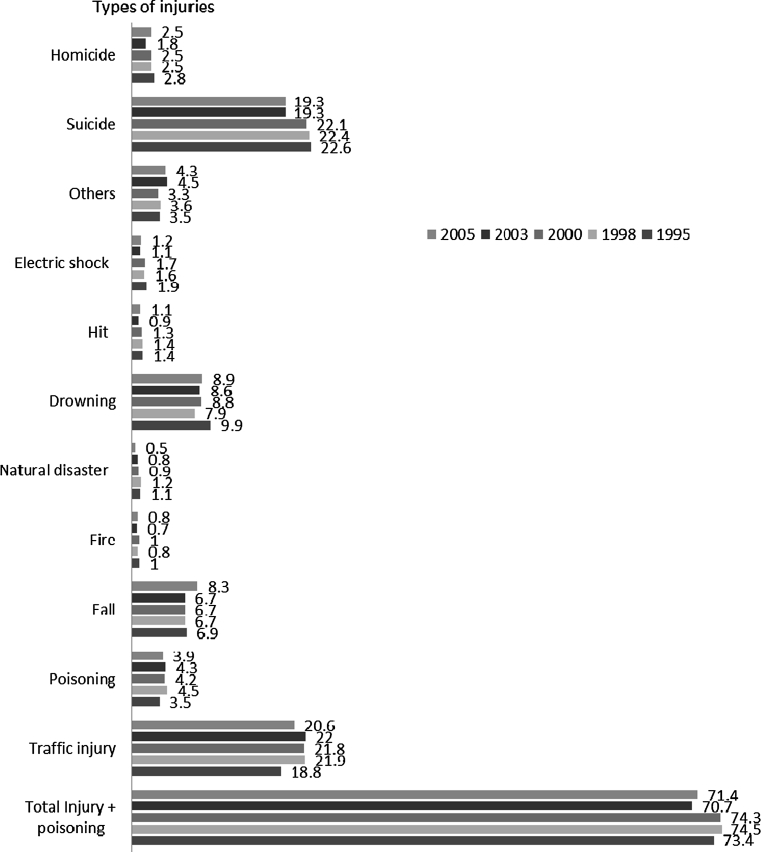
Estimated number of injury deaths in China (per 10,000 people) during 1995–2005

The causes of death in the Statistical Yearbook of China showed that in 2005 the injury mortality rate was 52.6 per 100,000 population (64.0 males and 38.4 females). The ratio of males to females was 1.7:1. In urban areas the mortality was 38.8 while in rural areas it was 59.0 (a ratio of 1:1.5). It was estimated, based on the current available data, that in China during 2005 the total number of deaths due to injuries was 732,000. Among them the main causes were traffic injuries, falls, drowning, and suicide. Those four kinds of injuries killed more than 550,000 people, constituting 75 % of the total deaths (Fig. 1) [18].

### Injuries and Economic Burdens

Injuries accounted for 17 % of the disease burden in adults aged 15–59 years in 2004 [19]. In China, if we remove the injury deaths, the life expectancy will increase by 1.2 years in city areas and 1.8 years in rural areas. The effects on life expectancy in rural areas are higher than on those in cities, reflecting the difference both in injury prevention and in the capacity for emergency management and medical treatment. The loss of life expectancy due to traffic accidents and suicide accounted for 43.8 % of the total and they were 0.3 years and 0.4 years separately. In China, injuries caused the largest amount of potential productive years of life lost (PYLL, 12.6 million years), more than for communicable or non-communicable diseases [16]. Injury-incurred PYLL account for one-quarter of the total deaths, similar to the total PYLL due to infectious, perinatal, and gynecological diseases in China [6].

During 2005, the direct economic burden of injuries reached 77.5 billion yuan (RMB), an increase of 4.9 times compared with 1993. The rise was faster than the per capita health spending over the same period (4.5 times). The indirect economic burden of the same year reached 275.1 billion yuan (RMB), an increase of more than 6.2 times the figure for 1993. This rate was higher than the rate of GDP increase (5.2 times). Over the 12-year period, urban China’s direct economic burden increased 5.2 times and its indirect economic burden of injuries increased 6.5 times. In the same time period (1993–2005) rural China saw a 4.7 times increase in the direct injury economic burden and a 6.0 times increase in the indirect injury economic burden [20]. However, some injury events involve many people with an influence on and harm to the whole society, including much larger unaccounted social and economic effects than those on individuals and families.

In a study in ShanDong Province 13,919 residents in rural areas were investigated. The results showed that injuries and poisoning ranked as the first reason for hospitalization in rural areas, accounting for about 20 % of inpatients. Once an injury event occurred, the amount of money the rural residents needed to spend on hospitalization was about the average net income in a 2.3-year period, which is much higher than for other diseases [21]. Since China is such a large country, whether in terms of population or geographic area, the mortality of injuries differs a little in each province, including the economic burden of injuries [22]. For example, injury mortality in Guangdong Province in rural areas was 31.8 and in urban areas 12.4 per 100,000 population (1999–2003); in Hubei Province it was 58.5 and 31.6; and in Beijing, the capital of China, the injury mortality was 38.2 per 100 thousand population [23, 24].

### Causes and Risk Factors of Injuries

#### Traffic Injuries

Before 1955 there were fewer than 10,000 traffic accidents each year in China and the number of deaths was fewer than 1,500. In 1975, compared with 1955, the traffic accidents increased 9 times and the number of deaths increased 17 times. Since the 1980s the number of traffic deaths has increased at a rate of 10 % each year and after the 1990s the trend was still growing but the growth rate decreased slightly [25].

The Chinese national road traffic accident data from 2006 to 2008 shows a decrease in the number of crashes, deaths, and injuries and the mortality by population, vehicles, and road mileage. The number of deaths per crash increased during that period. Most of the injured people were male and aged between 21 and 45 years, with an increasing proportion of people aged over 60. The majority of the victims were passengers, motorcyclists, and pedestrians. Road accidents mainly occurred at night, between 7 and 8 p.m. being the peak hour, and most deaths occurred between 3 and 5 a.m. Road traffic injuries were caused mostly by motor vehicles, mainly passenger cars, motorcycles, and vans. Electric bicycles were the main type of non-motor traffic vehicles that caused crashes. The number of crashes and casualties showed an upward trend every year. RTI crashes caused by motor vehicle driving were due to law violations. The five leading causes of RTI crashes were: speeding, not giving way as required, driving without a license, retrograde driving, and driving under the influence of alcohol [26–29]. The number of motor vehicles in China only accounted for 1.6 % of the total number in the world; however, the death rate due to car accidents accounted for 14.3 % of the total deaths from the same injuries in the world and the trend is still increasing [30]. Research investigating 1,340 drivers about their unsafe driving behaviours during the last month showed that the frequency of unsafe driving behaviours in rural areas (88.3 %) was much higher than in urban areas (57.8 %) and the figure for male drivers (76.4 %) was much higher than for female drivers (66.7 %). The percentages of driving without a seatbelt, drink driving, fatigue driving, and driving without a license were 35.2, 12.2, 9.3, and 26.6 %, respectively [31].

#### Fall

In some big cities falls have become the leading cause of injury deaths among elderly adults in recent years [32, 33]. For people aged over 70 years, the top 3 causes of injury deaths were traffic accidents, falls, and suicide. Males had higher suicide mortality than females, accounting for 33.1 % injury deaths of males, followed by traffic accidents and falls. In females the percentages of suicide, falls, and traffic accidents were 30.9, 16.8, and 15.4 %, respectively [34].

In Shanghai Xuhui district, the mortality of falls among the elderly population (>60 years) was 56.4 per 100,000 population, accounting for 64.9 % of the total deaths. In different age groups the death causes were different. In the 70–79 age group and the 80 and over age group falls were the leading cause of death [35].

For elderly adults, over 50 % of falls occurred at home and most falls happened at the same height level. For fall injuries occurring outside the home, the most common places of incidences were roads and streets, farm markets, transport vehicles, and public places. The risk factors for falls among elderly people included high family income, distraction, lack of sleep, and dissatisfaction with family life and relationships [36]. Living alone, a low level of walking and daily activity capacity, and mental damage were the main risk factors for falls among elderly adults. Fall injuries happened more at home among the elderly living alone [37, 38]. For children in big cities falls are also the leading cause of injury deaths and hospital visits. In Dalian, for children aged from 0 to 14 years, fall injuries accounted for 45.3 % of hospital visits [32].

In 2003 a survey was carried out in three big cities: Beijing, Shanghai, and Guangzhou. The results showed that for children aged 0–14 years, the most common injury resulted from falls. The incidence of falls was 5.7 %, which accounted for 34.7 % of unintentional injuries among children [39].

Risk factors related to falls among children included poor living environments, a small living space, a poor family economic situation, parents being labour-intensive, adults having little time to play with children, scatterbrained children, an adventurous personality, quick mood changes, and liking to play in high places [40, 41]. For children in kindergarten, the materials of the floor and the playground condition were significantly related to the incidence of falls [42].

#### Drowning

In China the main cause of injury deaths for children aged 0–14 years is drowning, which accounts for over 50 % of deaths in this age group. For boys, almost 60 % of deaths were due to drowning [43]. A survey among middle and high school students in 18 provinces in China showed that 52.1 % of the investigated children had reported swimming, 12 % had swum in an unsafe swimming place, and 2 % had experienced drowning. Boys had higher rates of domination than girls. Big cities with coastal areas and water-rich provinces had a higher reported swimming rate. The proportion of swimming in safe swimming places was higher in economically developed cities and provinces than in underdeveloped cities and provinces. Affluent areas had a lower reported drowning rate [44]. In Haidian District, Beijing, death reports and death causes showed that from 1974 to 1991 the leading cause of mortality among primary and middle school students was drowning, with a mortality rate of 117.8 per 100,000 students [18]. Among 181 drowning deaths, 176 cases occurred when swimming (97.2 %) in waters not specified for swimming. Only 5 deaths occurred at swimming pools [45]. In a case–control study in Guangxi Province the results showed that falling into water was the leading cause of drowning deaths for children in rural areas, where most drowning deaths occurred on-site [43]. The majority of drowning occurred in warm seasons, summer and autumn. Four significant risk factors were mothers’ drinking (OR = 13.3), nobody with knowledge of and skills in first aid for drowning children in the village (OR = 10.5), caregivers being out of their wits when children were drowning (OR = 6.4), and children often playing close to the water (OR = 2.0). Four significant protective factors were taking a swimming course (OR = 0.04), being watched by a caregiver when swimming (OR = 0.034), having a caregiver in a good health condition (OR = 0.030), and communication with parents (OR = 0.5) if necessary [43].

For non-fatal drowning, the events mainly occurred in rivers or ponds (71.1 %), followed by swimming pools (14.9 %) and wells (2.2 %). The activities engaged in when drowning occurred were swimming (41.8 %), diving or scuba diving (15.2 %), playing in the water (10.8 %), and walking or running or washing on the waterside (7.1 %). The main causes were falling into water (23.8 %), poor swimming skills (22.9 %), diving or scuba diving (16.1 %), playing in the water (12.0 %), and cramp (7.4 %) [46].

#### Suicide

Suicide has long been identified as one of the major injuries in China [30, 47, 48]. Suicide was the leading cause of injury deaths among elderly persons aged over 60 [35]. Based on the data on death causes from the Chinese national disease surveillance system established using random sampling from 1990 to 2000, a total of 13,123 suicide deaths were reported: 924 cases in cities (492 males, 432 females) and 12,199 in rural areas (5,712 males, 6,487 females). In 1991 suicide was the first cause of injury deaths, accounting for 26.2 %. During 2000 traffic fatalities constituted the leading cause of death, while suicide was the second leading cause of death, accounting for 24.4 % of the total injury deaths [49].

The characteristics of suicide in the Chinese population differ from those in many other countries, with the female suicide mortality rate being higher than the male [47, 48, 50]. The ten-year average suicide mortality figure for males was 15.7 per 100,000 population, while for females it was 18.0. Suicide mortality in rural areas was 4 times higher than in city populations [49, 50]. Some scholars thought that this was due to the cultural differences, while others considered that it was related to social development and social changes [47]. The average suicide mortality rate increased with age. The rate rose moderately before the age of 60 and rose sharply after that. Suspension and suffocation were the most common methods of suicide (57.1 %), followed by the consumption of pesticides (20.1 %) [51].

In China the suicide mortality in rural areas was higher than in cities. Cao et al. [52] reported that from 1990 to 1997 the suicide mortality in rural areas fluctuated between 22.5 and 29.1 per 100,000 population while at the same time in cities that rate was between 6.5 and 9.1. Suicide has been maintaining the first place in causes of death among the 15–65 age group in rural areas [53]. This may be due to several inter-related factors, such as differences between rural and urban areas, which have been envisaged; for example, in rural areas there is a relatively low level of economic development, a low mental health service level, and difficulty in obtaining an appropriate diagnosis and treatment for mental illness. In rural areas the first aid for pesticide poisoning is not as good as in city areas, which may lead to more deaths [54, 55].

### Safe Communities in China

The Safe Communities concept began its formal existence at the First World Conference on Accident and Injury Prevention held in Stockholm, Sweden, in September 1989. There are seven indicators for International Safe Communities which started to be in action from 2012 and before 2012 there were six indicators in which indicator 4 was not included [56]:

1. An infrastructure based on partnership and collaborations, governed by a cross-sectional group that is responsible for safety promotion in its community;
2. Long-term, sustainable programmes covering both genders and all ages, environments, and situations;
3. Programmes that target high-risk groups and environments, and programmes that promote safety for vulnerable groups;
4. Programmes that are based on the available evidence;
5. Programmes that document the frequency and causes of injuries;
6. Evaluation measures to assess their programmes, processes, and the effects of change;
7. Ongoing participation in national and international Safe Communities networks.

A community can be defined as a delineated geographical area, groups with common interests, professional associations, or individuals who provide services in a specific location [56].

The first designated Safe Community in China was the Youth Park Community of ShanDong Province in 2006. So far 35 communities have successfully fulfilled the six indicators and become members of the International Safe Community Network in mainland China. Those communities are located all over China, including Beijing, Shanghai, Shenzhen, Dalian, Hebei Province, Shanxi Province, and Shandong Province.

Those communities could be divided into two types: at this moment the first one is urban communities, which are located in a city; and the second one is enterprise-dominated communities, which means that the community is part of an enterprise or company or the community itself is the enterprise or company, such as the Luan Group, which consists of a production area and a residential area. So far 3 of the 35 communities are enterprise-dominated communities and the others are urban-setting-based communities. Since the Safe Community concept was introduced into mainland China, local governments have been paying increasing amounts of attention to safety promotion and injury prevention at the community level and some of them have put the Safe Community project into the government working plan. In China a Safe Community is more likely to follow a kind of top-down model, which means that the government takes the main responsibility for initiating, promoting, coordinating, and funding the project.

Based on reviews of the application reports for becoming members of the International Safe Community Network of those designated Safe Communities, the programmes focused more on unintentional injuries, including workplace safety, falls among the elderly, traffic injuries, school safety, home safety, etc. Education and environmental renovation and improvement were the main strategies used in terms of injury prevention. Some communities have carried out and established very good injury surveillance systems based on their own resources, but some failed to work in a very efficient way or even lacked essential information in this regard [56].

## Discussion

The community-based approach to injury prevention programmes was developed in the 1980s and has since become an essential component of injury prevention [57]. This approach is based on the premise that the community is both the source of safety problems and the means by which solutions are achieved. Community-based programmes are characterized by collaboration among different community sectors and the involvement of community members to define the safety problem and find solutions [58, 59].

The Safe Community programme in China may have covered some aspects of injury prevention and safety promotion. In Safe Community programmes in China, children, the elderly, cyclists, and pedestrians are the prioritized target groups. However, other vulnerable groups, such as migrant workers, motorcyclists, students, players, and farmers, should gain the necessary attention from the Safe Community perspective. As the evidence describes, Safe Communities in China have not emphasized multi-focused and multi-sectoral programmes to have effects on specific injury prevention programmes for specific target groups.

Concerning the injury situation, the social economic and development status, and Safe Community development in China, the following four issues should be taken into consideration.

### Establish and Strengthen the Policy and Regulations in Terms of Injury Prevention at the National Level

Since the 1950s, the national and local governments in China have gradually developed a series of injury-related prevention and control policies, laws, and regulations, including work safety, road safety, school safety, and so on. The implementation of those laws and regulations has led to a certain degree of decrease in injury incidence, the severity of injuries, and social and economic losses. However, those injury prevention policies were not attempted as a mainstream policy under a specific agenda. In general injuries still contribute greatly to residents’ illness and death in China and there is a wide gap between what needs to be done and what has already been done in terms of injury prevention [60, 61].

Even though the Safe Communities programme is conducted at the community level, the overall policies, regulations, strategies, and planning should be set up at the central government level. The collaboration and coordination of different government departments need to be arranged overall by the central government as well. The injury prevention and control work must be formed under the guidance of the government. At the same time social attention and community participation are also very necessary. It is important to transfer the experts’ behaviours into government behaviours [4, 60].

The most important issue is to work on the national plan for injury prevention and control, which should be part of the national public health plan [62]. The planning process of the national injury prevention system should be accelerated. The supporting environments need to be established, priority areas need to be determined, and the departments taking primary responsibility need to be appointed. Moreover, injury prevention and control should include goals and specific objectives in terms of each priority area, strategy, and action plan. The Chinese Government functions very strongly in organization, coordination, and cooperation. By taking advantage of this tradition, Safe Communities should work together with at least those departments or sectors that include public security, transportation, health, education, occupational safety, and many others.

### Create a System to Involve Professional Organizations and Personnel in Projects

Situational awareness is very important for creating good injury prevention programmes. This means that to achieve good injury prevention programmes it is necessary to have a good understanding of the injury situation, risk factors, and protectors in terms of injury occurrence in the community [5]. It is also very important to undertake appropriate and scientific evaluation of the programmes. From the review of application reports, we found that this is the weakest point in terms of the development and evaluation of injury prevention programmes at the community level.

Recently the WHO pointed out that measures of effectiveness should cover the short-term, the medium-term, and the long-term evaluations. It is particularly important to establish baseline measurements before an intervention is implemented [63]. Those duties and activities need the involvement of professional organizations and personnel. Without their professional knowledge and skill, it is impossible to fulfil those functions.

At the national level, the injury definition, classification, surveillance sample size, coding, data collection, establishment of a database, evaluation, outcome assessment, analysis, and reports need to be standardized. In China it is possible to obtain some injury data from certain ministries, such as Public Security, Transportation, Occupational Safety, and Health. However, a complete and comprehensive report system in terms of injury mortality and morbidity is lacking. Another concern is that, being a very big country, China must be very careful about using the national data to develop priority projects for particular communities.

We would like to say that academic involvement in Safe Communities is a win–win situation. Communities can obtain professional advice, support, and guidance. On the other hand, communities can act as a test field for researchers to create, improve, and promote academic study in relation to injury prevention and safety promotion. Finally, safe communities altogether will contribute to the national and global injury prevention and safety promotion.

### Consider the Economic Development Status

In terms of injury mortality in China, we found that rural areas had a higher rate than urban areas, the western part of China had a higher rate than central China, and the eastern part of China had the lowest injury mortality rate [34]. These differences are also reflected by the economic development of different parts of China. The eastern part of China is the most economically developed, the central part is the second, and the western part is relatively the most backward. Meanwhile, in general economic development in cities is significantly better than in rural areas.

In China there were differences both in the injury death spectra and in the death levels between urban and rural areas. The suicide mortality rate in rural areas was three times that in urban areas and ranked as the first injury cause of death [48]. Although in recent years in rural areas traffic injury mortality has been increasing, suicide mortality was still higher than traffic injury mortality. Drowning was the first cause of injury death among children in rural areas. The top four causes of injury death in rural areas were suicide, traffic accidents, drowning, and unintentional poisoning. Meanwhile, in urban areas the top four causes of injury death were traffic accidents, suicide, falls, and unintentional poisoning. Drowning ranked as the sixth cause of death in urban settings. The living environment and lifestyle lead to the differences in the injury death spectra between urban and rural areas. Safe Communities have a major criterion of injury prevention for the specific target groups based on surveillance. The existing Safe Communities in China should deliberately focus on this criterion to eliminate the above-described inequalities in injury control.

We would like to mention that the cost-effectiveness of the Safe Community programme must be considered throughout the project. Regarding the Safe Communities themselves, these multifaceted programmes typically address multiple risk factors in multiple settings, with the aim of maximizing the programme’s effects throughout the community [5, 57].

Some successful worldwide experience should be given special attention. For example, in terms of traffic injuries, in general, since the 1960s/1970s there has been a decrease in the numbers and rates of fatalities in high-income countries. The reductions in road traffic fatalities in high-income countries are attributed largely to the implementation of a wide range of road safety measures, including seat-belt use, vehicle crash protection, traffic-calming interventions, and traffic law enforcement. Of course, local knowledge needs to be fed into the implementation of local solutions, in which Safe Community programmes play a vital role [5].

### Intentional Injury Prevention Should Receive Greater Attention

In China, the main causes of injury mortality (in descending ranking) are: suicide, traffic accidents, drowning, falling, poisoning, homicide, burns and scalds, and iatrogenic injury [6]. However, suicide is yet to receive the necessary attention from the authorities. The lack of sensitivity to suicide issues by the government and society, the lack of a national death registration system and a monitoring system for suicide attempts, low capacity in terms of identifying mental disorders by health-care professionals, difficulty in coordination of multi-agency work, and lack of tools for effective assessment of the psychological situation are also important [64]. The WHO noted that suicide prevention efforts will be ineffective if they are not set within the framework of large-scale plans developed by multidisciplinary teams, comprising government officials, health-care planners and health-care workers, and researchers and practitioners from a variety of disciplines and sectors [65]. Moreover, there is an important cultural context for successful suicide prevention efforts as well. The necessary cultural norms include: (1) positive attitudes toward help-seeking, (2) an accurate understanding of mental health and mental illness, and (3) an emphasis on interdependence and interconnectedness [66].

Those components, whether relating to multi-sector collaboration or to the local cultural context, have already been involved in Safe Community initiatives. The key points for the communities are: (1) recognize and understand the suicide issue and that it is a serious and harmful event that is preventable, (2) as long something is done, there should be an outcome, and (3) find partners to work together.

In reality, China is still at a very early stage of injury prevention and control. Comprehensive and integrated prevention strategies and methods must be further strengthened and developed. Important safety issues, such as a seat-belt law, safe highway design, child seats inside cars, safe, pedestrian-friendly footpaths, life-saving devices in public swimming areas, etc., should also be emphasized. The sociological structure of China, including rapid economic growth and development itself, indicates the need to focus on inter- and intra-sectoral solutions to injuries on the primary level. Safe Communities as a policy issue have provided an effective platform for developing and carrying out injury prevention and safety promotion strategies and policies in China. The evidence comes from a few areas in China, which has vast geographical, social, and economic diversity. However, until now, at the national level China has lacked overall plans and guidelines to coordinate Safe Community-related work and research, including state financial support.

There is a dearth of peer-reviewed articles on Safe Communities in China. The current paper suffers from a lack of sufficient articles in the Chinese context. On the other hand, grey materials galore are available on Safe Communities in China. In this context, another study examining the long-term effectiveness of Safe Communities and other safety criteria is warranted. As far as policy issues are concerned, the current paper has utilized all the relevant articles and materials available on the Internet. However, after reviewing the field, we can recommend more scientific articles on the issues under discussion. Due to very few published scientific papers studying the effectiveness of Safe Communities with data based on a comparison of before and after the designation of a Safe Community, it is difficult to show evidence to demonstrate the effectiveness of Safe Communities in China. However, this evidence is very important. It can be the basis for why we should further promote the idea to prevent injuries at the community level. Safe Communities do not mean that each community works separately. Through the efforts of each community, they can share their experiences, they can advocate to the higher authorities to put more emphasis on a specific injury problem, and they can provide collective evidence to prove the effectiveness of safe community activities. Though the study has focused entirely on China, the findings and arguments can be utilized in other developing countries.
